# Supplementary figures and images for: Hospital length of stay: A cross-specialty analysis and Beta-geometric model
Source: PLoS One. 2023 Jul 13;18(7):e0288239. doi: 10.1371/journal.pone.0288239 (PMC10343164; doi:10.1371/journal.pone.0288239)

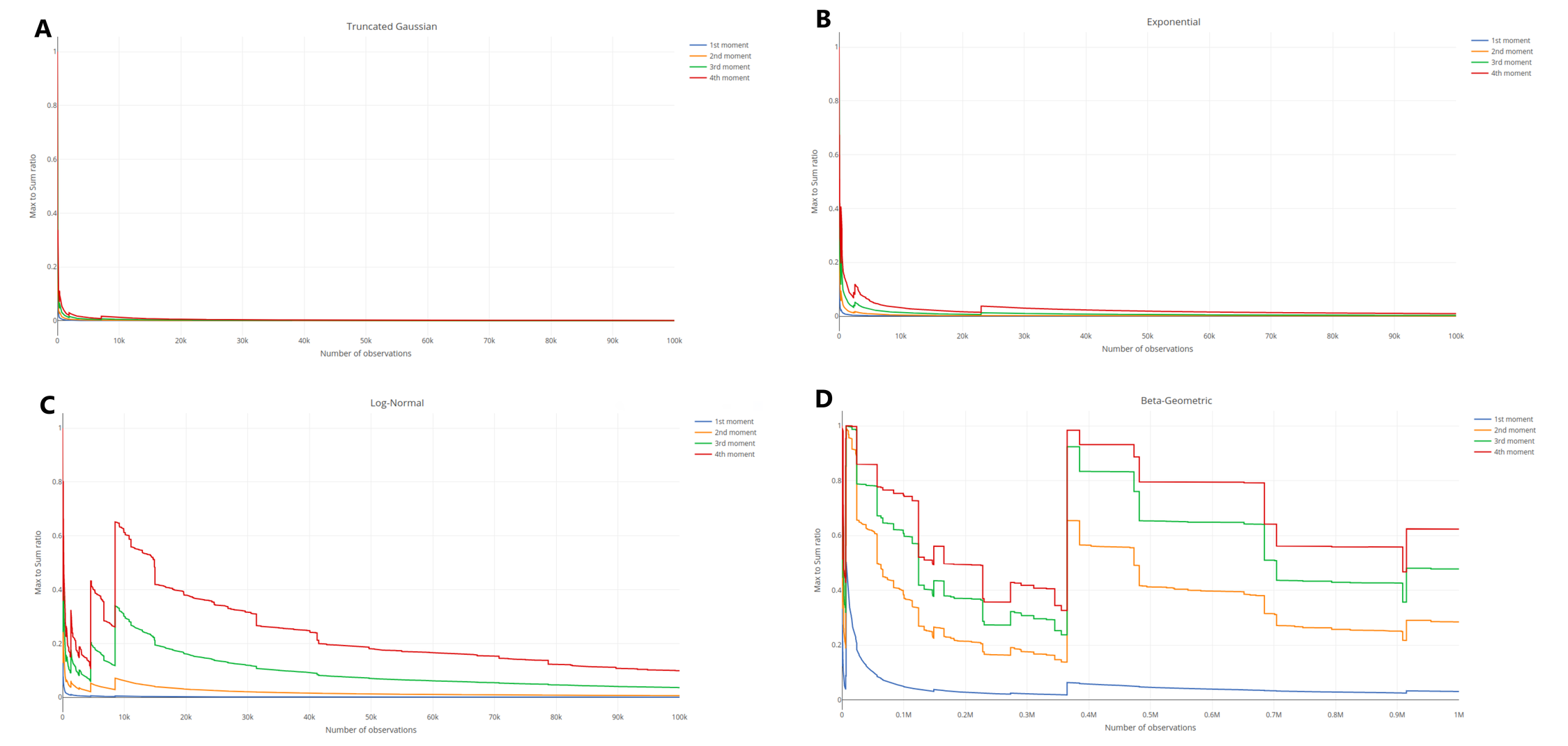

Supplement: S1 Fig — (A) Maximum to Sum ratios for a Gaussian. (B) Maximum to Sum ratios for an Exponential. (C) Maximum to Sum ratios for a Beta-Geometric with α = 1.3 and β = 3. (D) Maximum to Sum ratios for a Beta-Geometric with α = 1 and β = 3. (TIF) [file pone.0288239.s001.tif]

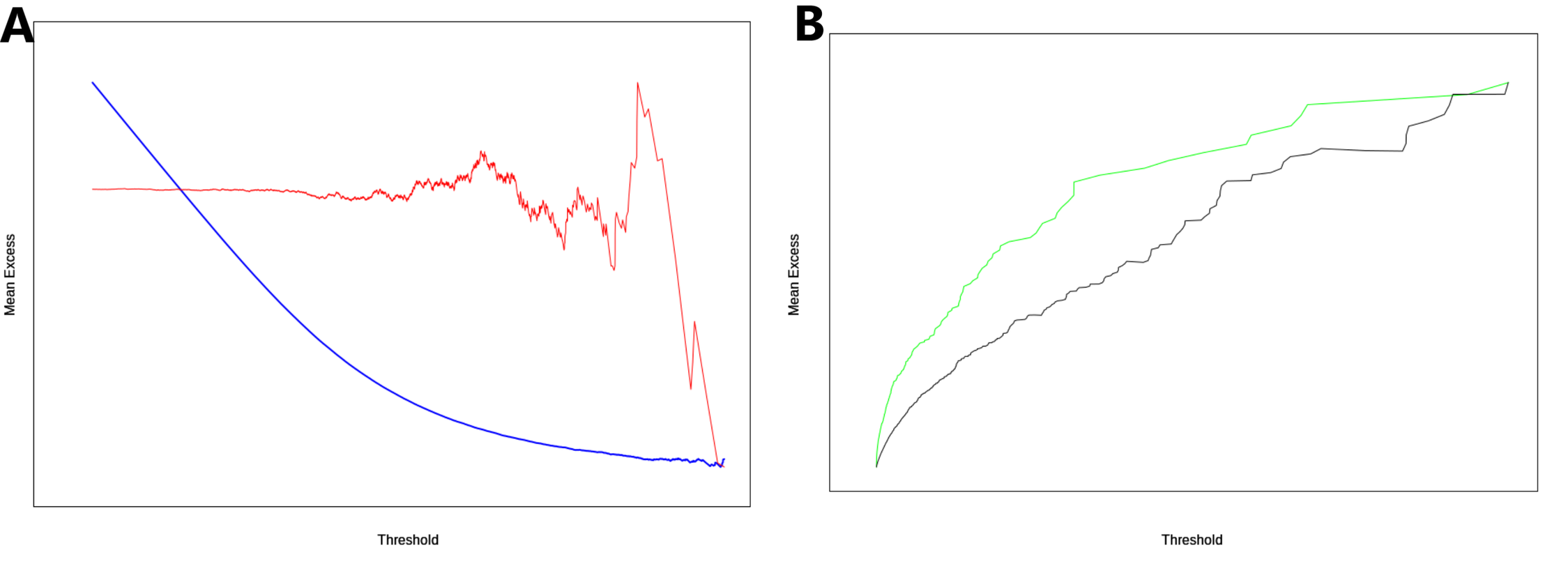

Supplement: S2 Fig — (A) Mean Excess Functions for a Gaussian and Exponential. (B) Mean Excess Functions for a Pareto and Sub-Exponential. In S2A Fig, we plot the Mean Excess function of a truncated Gaussian (blue line) of mean 10 and variance 3 and Exponential (red line) of rate.1. Note that the chaotic perturbations at the extremity of the red curve are just the effect of the finite sample bias, i.e. the fact that points for very high order statistics in the plot are the result of very few observations [44]. S2B Fig present the mean excess functions of the exponential of the previous Gaussian, i.e. a Log-normal distribution (green line), as well as a Pareto distribution of shape parameter.2. The axis labels have been voluntarily omitted to fit both curves within one plot and highlight the monotonic shape of the functions rather than specific values. (TIF) [file pone.0288239.s002.tif]

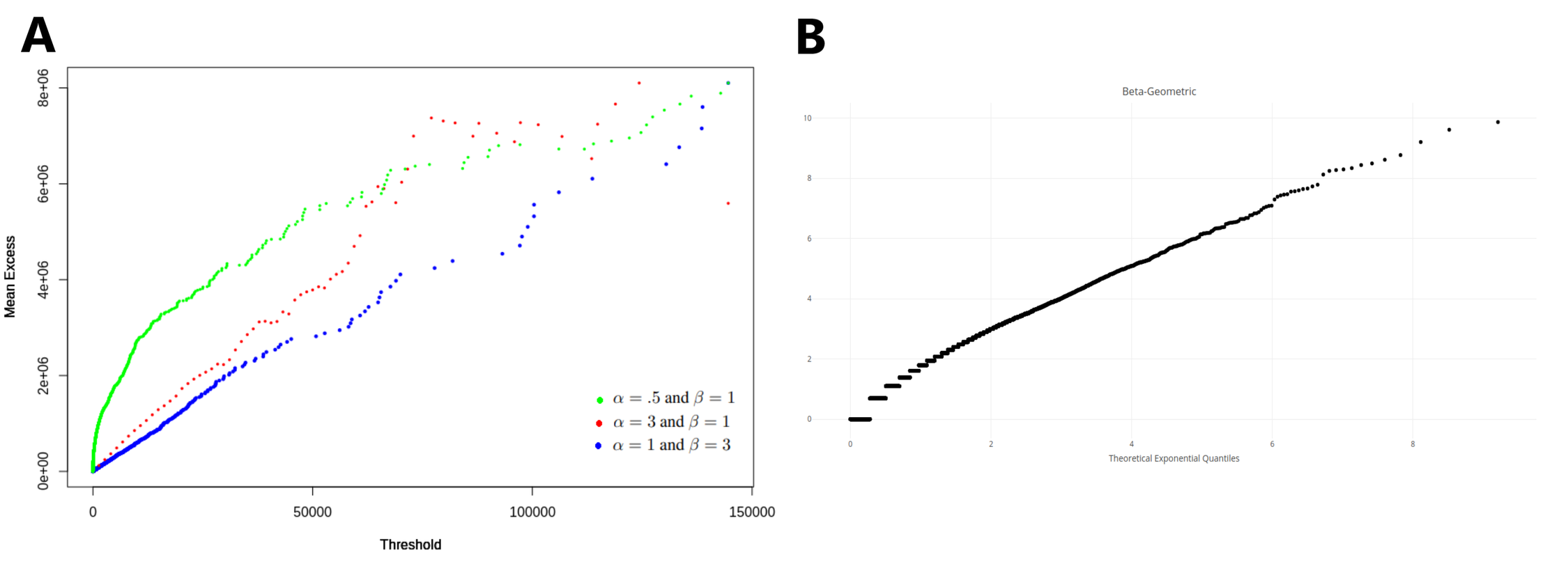

Supplement: S3 Fig — (A) Mean Excess Functions for Beta-Geometric distributions. (B) Q-Q plot for a Beta-Geometric. We have generated Monte Carlo samples of size 106 of Beta-Geometric random variables. As can be seen in S1D Fig, which represents the Maximum to Sum ratios for a Beta-Geometric with α = 1 and β = 3 the mean and higher moments do not converge. S3A Fig presents the Mean Excess function for a Beta-Geometric with α = 1 and β = 3 (blue points), α = 3 and β = 1 (red points), and α =.5 and β = 1 (green points). We can observe, the strictly increasing nature of these functions. Moreover, a log-transformed Pareto random variable is notoriously exponentially distributed. Hence a comparison of the theoretical quantiles of an Exponential random variable with those of the log-transform of empirical data is the basis of a visual Pareto test, known as the QQ (Quantile-Quantile) plot, Cf. Section, Gini Index and Lorenz Curves of [68]. A linear pattern indicates that the empirical data belongs to a generalized Pareto distribution and confirms heavy tails. S3B Fig presents a QQ plot for a 106 Monte Carlo sample of a Beta-Geometric with α = 1 and β = 3, in which linearity can be observed. In addition to the previous remarks concerning the non-convergence of moments and the increasing Mean Excess function, this observation makes us conclude that the Beta-Geometric can exhibit the behavior of a generalized Pareto model [69]. (TIF) [file pone.0288239.s003.tif]

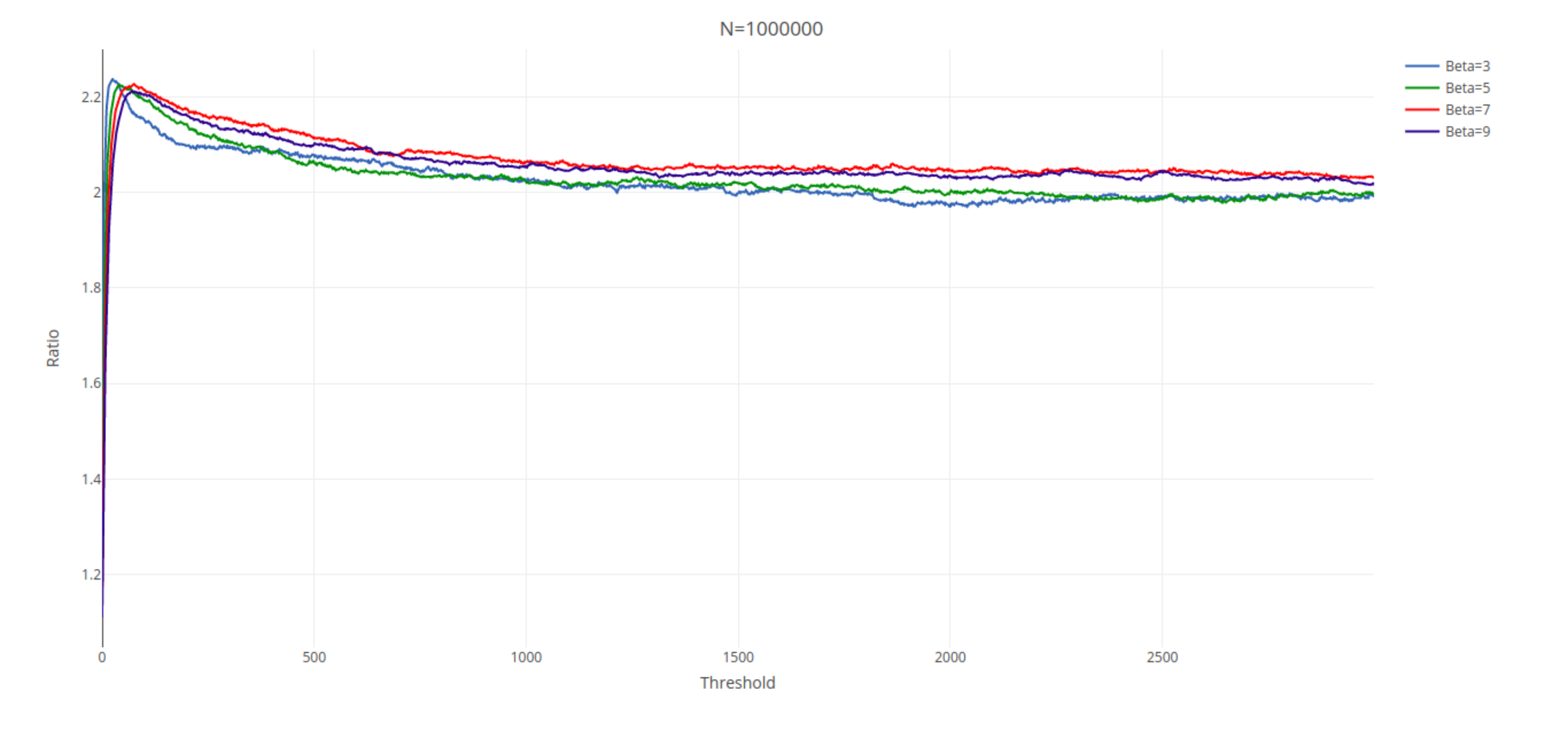

Supplement: S4 Fig — Beta-Geometric Distribution: The empirical limit of the ratio 1-F(2)(x)1-F(x) for α = 1.1 and β ∈ {3, 5, 7, 9}. Though the focus of this paper does not lie in the theoretical study of random variables, it can be empirically verified that the tail of this random variable is at least heavy enough to belong to the subexponential class for some values of α and β that result in even thinner tails than the values that seem adequate to model LOS in the dataset at hand. We have generated two random samples of 106 observations each from two identical Beta-Geometric distributions with α ≥ 1.1 and β ≥ α and verified the property defining subexponentiality. (TIF) [file pone.0288239.s004.tif]

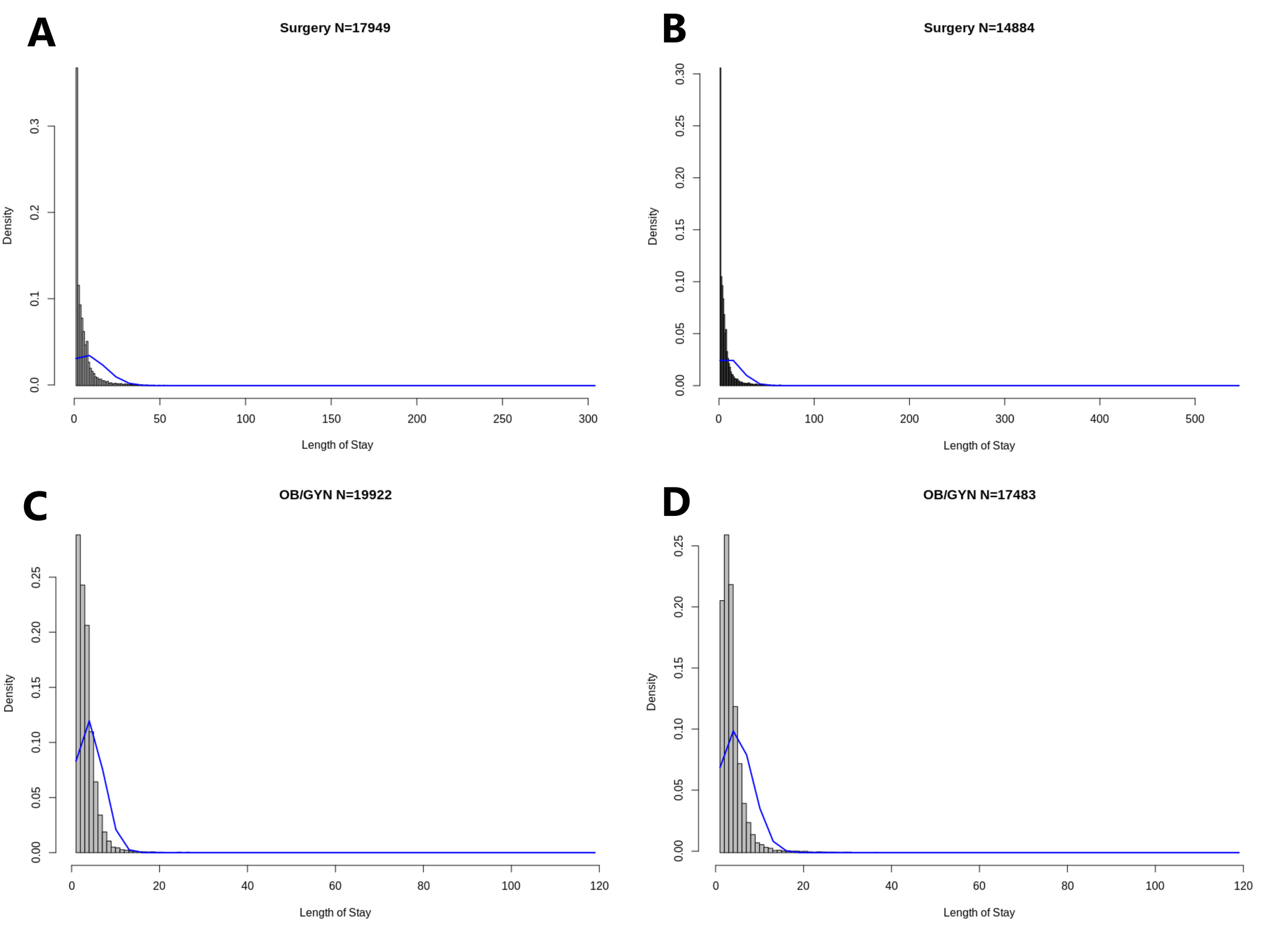

Supplement: S5 Fig — (A) Histogram of LOS per admission in Surgery. (B) Histogram of LOS per admission in Obstetrics and Gynaecology. (C) Histogram of LOS per patient in Surgery. (D) Histogram of LOS per patient in Obstetrics and Gynaecology. (TIF) [file pone.0288239.s005.tif]

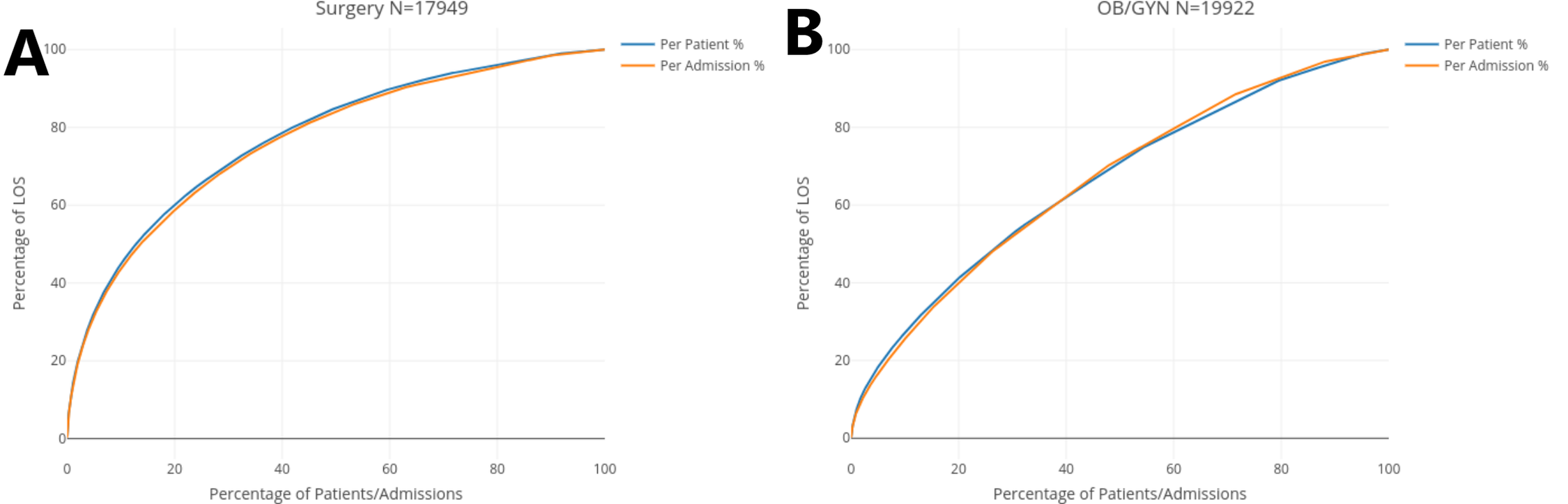

Supplement: S6 Fig — (A) Reversed Lorenz curve of LOS in Surgery. (B) Reversed Lorenz curve of LOS in Obstetrics and Gynaecology. (TIF) [file pone.0288239.s006.tif]

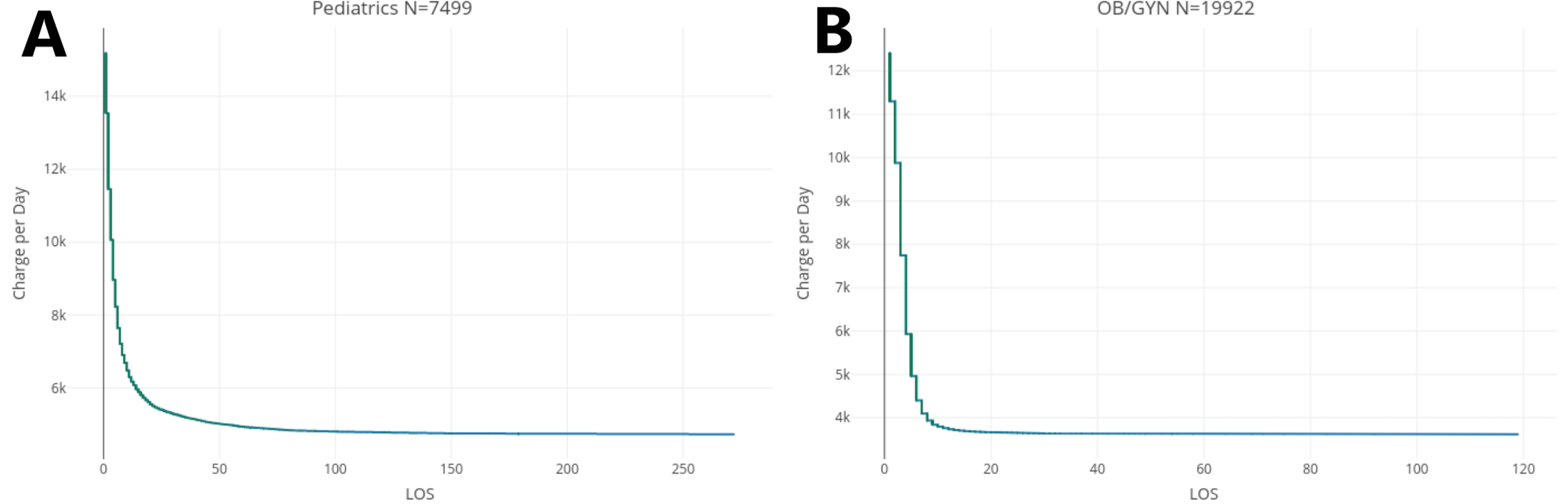

Supplement: S7 Fig — (A) Average charge per day in Pediatrics. (B) Average charge per day in Obstetrics and Gynaecology. (TIF) [file pone.0288239.s007.tif]

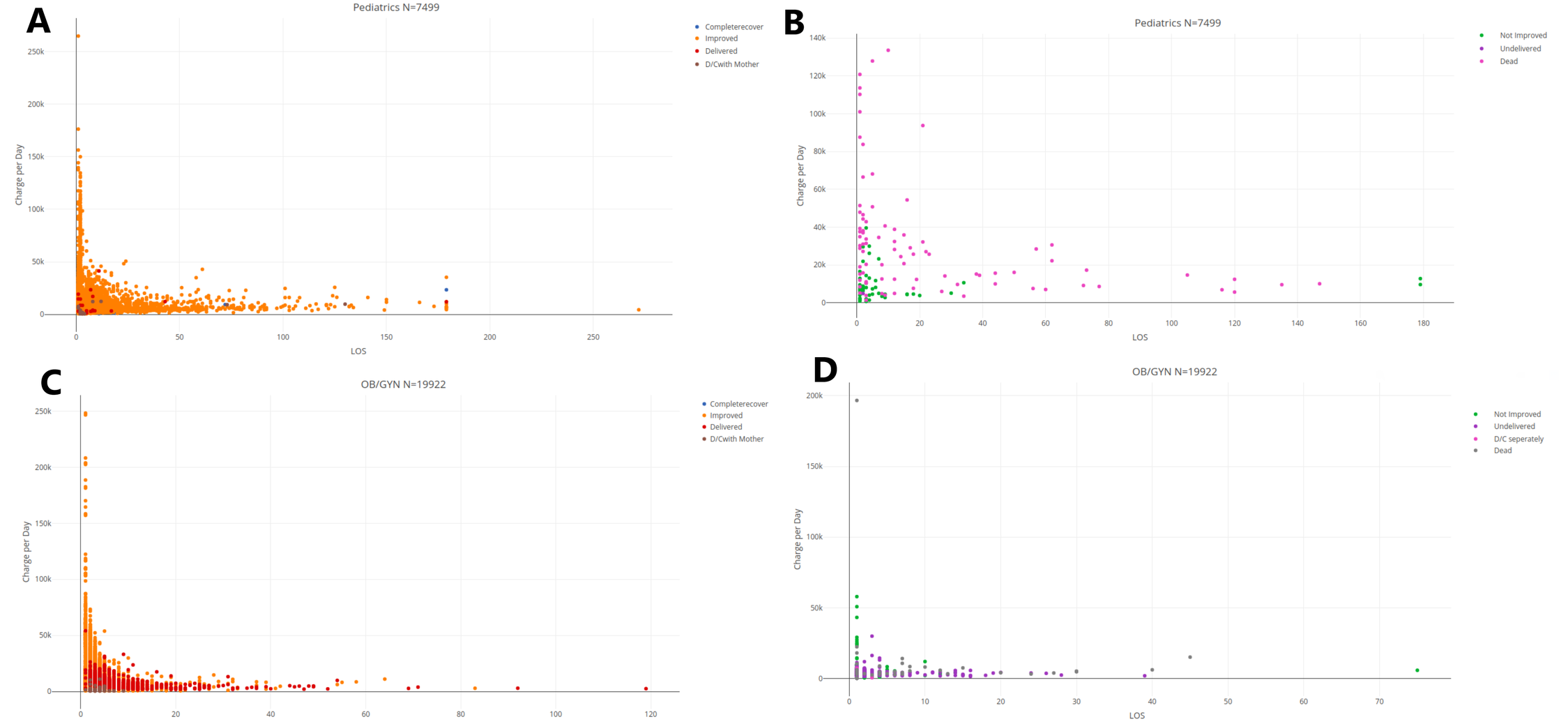

Supplement: S8 Fig — (A) Charge per day for positive discharge status in Pediatrics. (B) Charge per day for negative discharge status in Pediatrics. (C) Charge per day for positive discharge status in Obstetrics and Gynaecology. (D) Charge per day for negative discharge status in Obstetrics and Gynaecology. (TIF) [file pone.0288239.s008.tif]

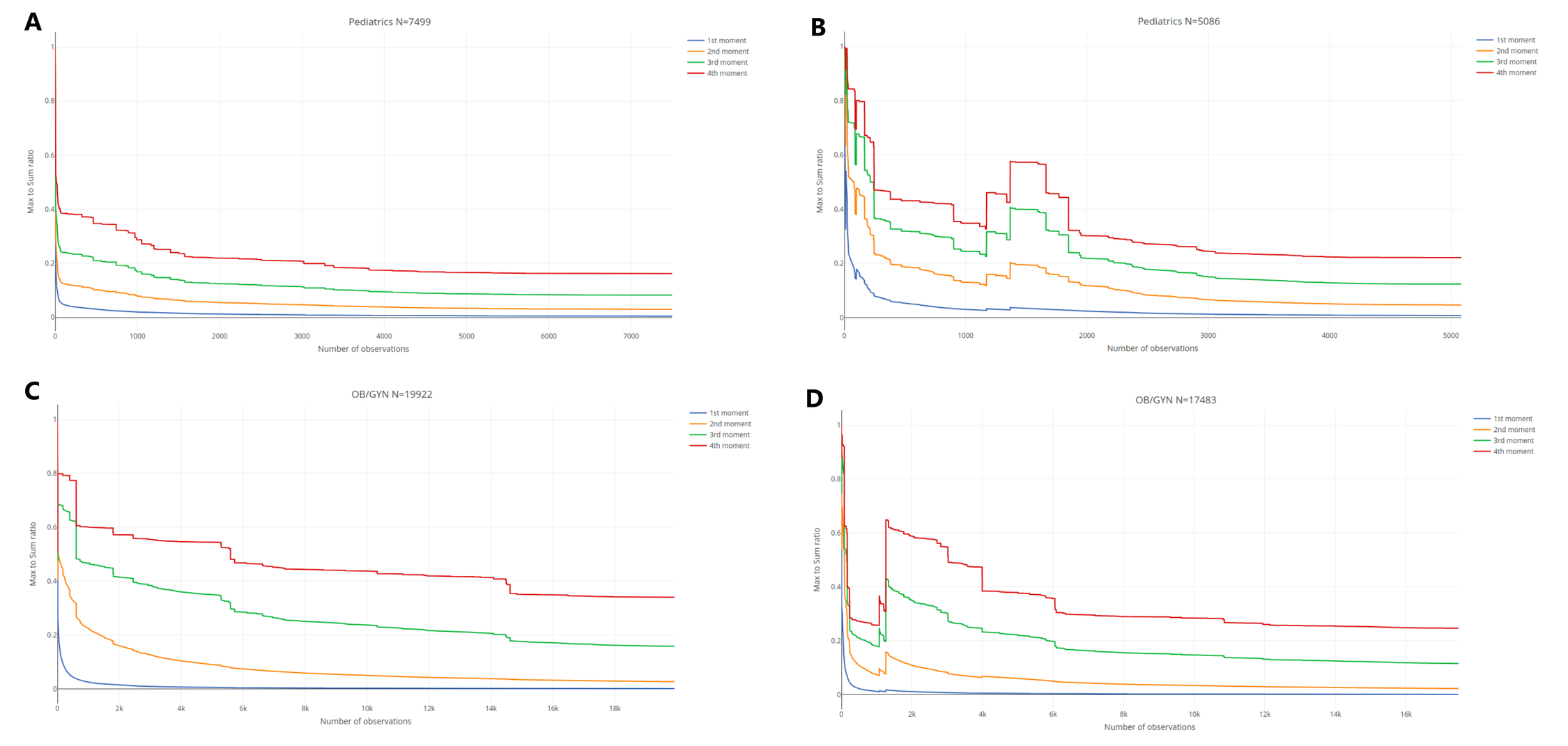

Supplement: S9 Fig — (A) Maximum to Sum ratios of LOS per admission in Pediatrics. (B) Maximum to Sum ratios of LOS per patient in Pediatrics. (C) Maximum to Sum ratios of LOS per admission in Obstetrics and Gynaecology. (D) Maximum to Sum ratios of LOS per patient in Obstetrics and Gynaecology. (TIF) [file pone.0288239.s009.tif]

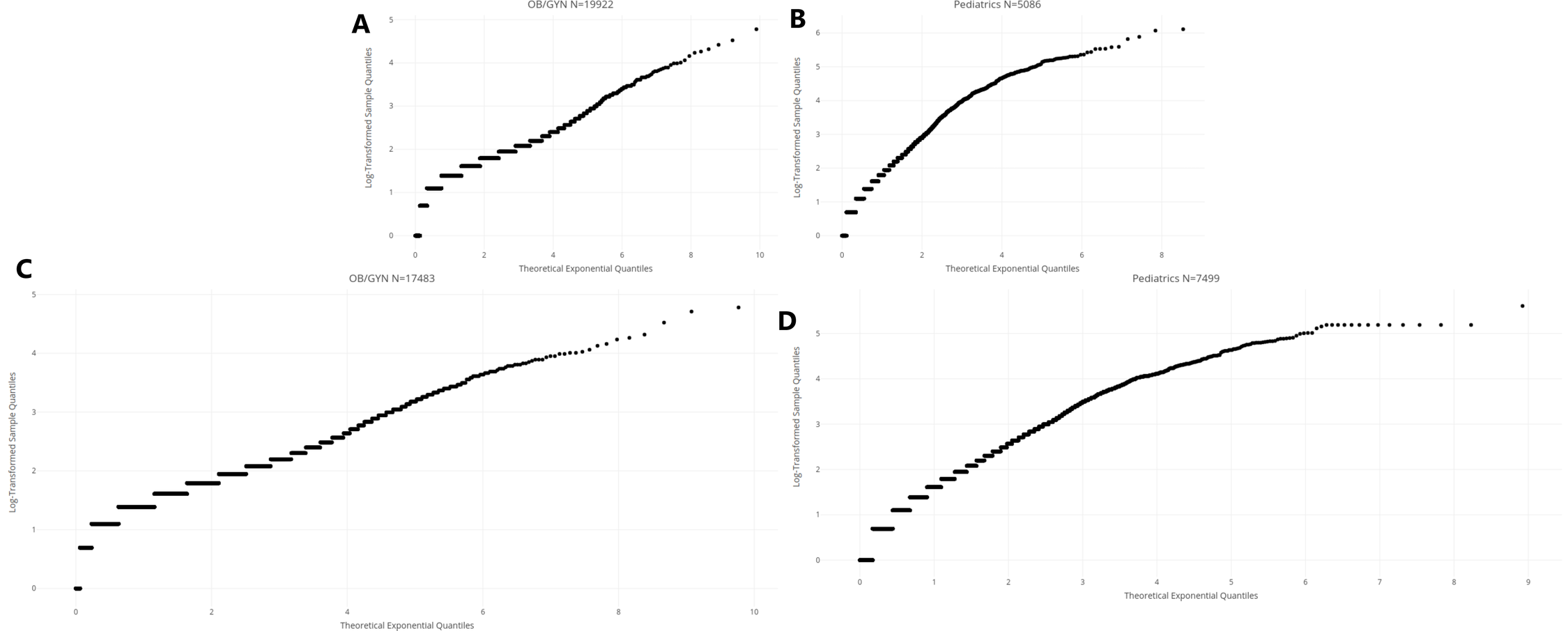

Supplement: S10 Fig — ((A) Q-Q plot of LOS per admission in Pediatrics. (B) Q-Q plot of LOS per patient in Pediatrics. (C) Q-Q plot of LOS per admission in Obstetrics and Gynaecology. (D) Q-Q plot of LOS per patient in Obstetrics and Gynaecology. (TIF) [file pone.0288239.s010.tif]

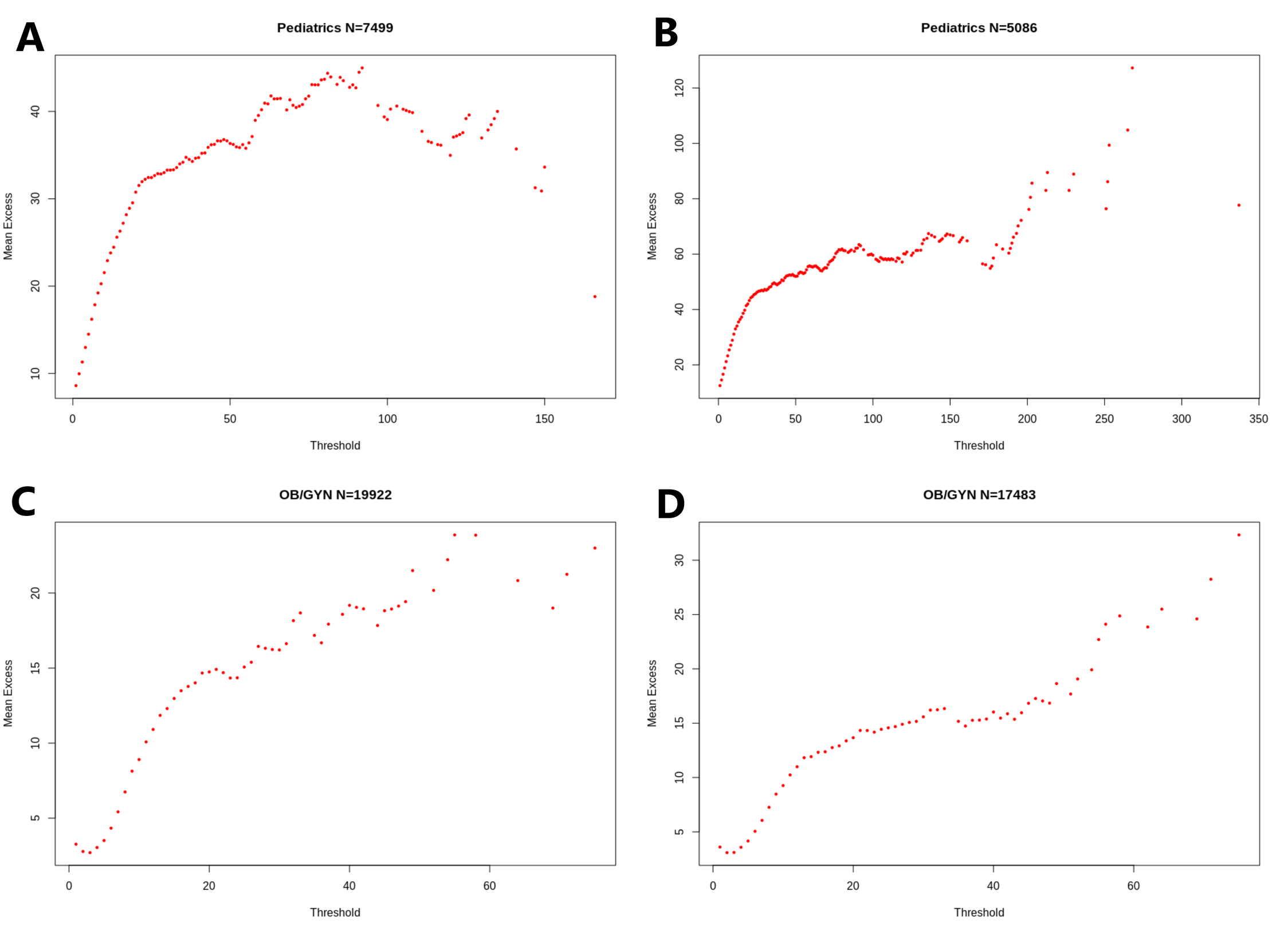

Supplement: S11 Fig — (A) Mean Excess function of LOS per admission in Pediatrics. (B) Mean Excess function of LOS per patient in Pediatrics. (C) Mean Excess function of LOS per admission in Obstetrics and Gynaecology. (D) Mean Excess function of LOS per patient in Obstetrics and Gynaecology. (TIF) [file pone.0288239.s011.tif]
