# Supplementary material for: Hospital length of stay: A cross-specialty analysis and Beta-geometric model
Source: PLoS One. 2023 Jul 13;18(7):e0288239. doi: 10.1371/journal.pone.0288239 (PMC10343164; doi:10.1371/journal.pone.0288239)
Supplement: Attachment — Submitted filename: Response to Reviewers.docx.pdf [file pone.0288239.s014.pdf]

Original Manuscript ID: PONE-D-21-29331
Original Article Title: “Hospital Length of Stay: A cross-Specialty Analysis and Beta-Geometric Model”
To: PLOS One Editor
Re: **Response to reviewers**

Dear Editor,
We are grateful for the review comments received. The structure of the paper has been revised according to the STROBE framework kindly recommended by Prof. Martins.

1. My main concern is related to the structure of the article. You did not follow the STROBE recommendations and the paper as it is very difficult to review.
Please look at the following article and try to follow a similar structure.
Shaaban, A. N., Peleteiro, B., & Martins, M. R. O. (2021). Statistical models for analyzing count data: predictors of length of stay among HIV patients in Portugal using a multilevel model. BMC Health Services Research, 21(1), 1-17.

Thank you for this comment. We have restructured the manuscript accordingly.

2. Have the authors made all data underlying the findings in their manuscript fully available?

The PLOS Data policy requires authors to make all data underlying the findings described in their manuscript fully available without restriction, with rare exception (please refer to the Data Availability Statement in the manuscript PDF file). The data should be provided as part of the manuscript or its supporting information, or deposited to a public repository. For example, in addition to summary statistics, the data points behind means, medians and variance measures should be available. If there are restrictions on publicly sharing data—e.g. participant privacy or use of data from a third party—those must be specified.
Reviewer #2: No

Please kindly note that raw LOS data for the 46,364 considered admissions have been made available in the file “LOS Data.xlsx”.

Kind regards,
The authors
