# Supplementary material for: Hospital length of stay: A cross-specialty analysis and Beta-geometric model
Source: PLoS One. 2023 Jul 13;18(7):e0288239. doi: 10.1371/journal.pone.0288239 (PMC10343164; doi:10.1371/journal.pone.0288239)
Supplement: Attachment — Submitted filename: Response to reviewers.pdf [file pone.0288239.s015.pdf]

Manuscript ID: PONE-D-21-29331
Article Title: “Hospital Length of Stay: A cross-Specialty Analysis and Beta-Geometric Model”
Re: Revision notes and response to reviewers

Response to Reviewer #1’s Feedback
We are very grateful for your nuanced feedback and agree with your concerns regarding the precision of decisions made based on our mathematical findings. We concur that the manuscript’s main focus is on fundamental statistical properties, specifically relating to the choice of the best fitting statistical model for LOS data, and less on managerial and clinical extrapolations which have not been sufficiently supported.

Moreover, we emphasize that study does not only propose a model for decision-making - the beta-geometric distribution discussed in Section "4.5. Modeling Length of Stay," but also offers a detailed analysis of the inherent properties of empirical Length of Stay (LOS) using Extreme Value Theory, a well-established area of statistics.

Reviewer #1: I reviewed the authors’ original submission and was invited to review this R2. I do not think that the authors have addressed decision-making. The authors list two decisions on lines 41 and 42, “How to align the hospital configuration to forecasted patient volumes?” and “What proportion of time should be allocated to different specialties in operating theatres?”. I think that the authors need to refer to specific decisions, with specific mathematics, that has been studied in detail and show that there are PRECISE decisions to be made with the mathematics. This was not done in the R1. Otherwise, if the authors prefer, say that this is basic science. Please appreciate that basic science is fine.
However, to point out how the authors are not being precise, line 42 refers to operating theatres, and yet there is no modeling for turnover times, lengths of surgical cases, etc. I am in no way recommending that be added, it would be out of scope. I use this example to highlight that the authors are not studying the allocation of operating room times. They have NOT done what they report was done (line 42) nor that I recommended.
We appreciate your highlighting the need to clarify the primary focus of our research and its broader context within the field. Strictly speaking, the main decision addressed by our study is indeed mathematical and is: "What is the most appropriate random variable to model hospital length of stay?" We have highlighted that this is an active area of research with our comparative literature review, summarized in Table 1. of the manuscript.

However, as you astutely point out, decision-making related to capacity planning involves other critical factors beyond LOS, including additional operational and clinical data that are not directly considered by our model. These decisions are also highly contingent on the specific resources, strategies, and constraints of individual healthcare facilities.

In response, in the revised “Decision-Making Scale” section, we emphasize more clearly that our research focuses primarily on the mathematical modeling of LOS, while acknowledging the

broader, multi-dimensional context within which this modeling occurs. With this revision, we aimed to clarify that our model serves as one piece of the larger puzzle of capacity management decision-making, rather than a comprehensive solution. Moreover, we limit our conclusions, throughout the paper, to the modeling scale that is supported by our empirical data (i.e. specialty departments).

In the R1, the authors added reference 21 for deciding about individual surgeries to be postponed. This is a specific decision, not conjecture. There was similarly another paper by the same authors on forecasting for individual hospitals by time series showing accuracy of the modeling (Cureus 2020;12:e10847). The topic does indeed differ from that of the authors. The authors explain in their section (added for R1) that the "scale of modeling" is different. This seems entirely reasonable assessment. I'm not recommending that the authors add more about this. But, use these examples that precise consideration of a decision at their higher "scale of modeling" means, please, being precise and showing that the dependent variables that they are including are sufficient for the decision.

In response to these observations, in the Section "Scope of Decision-Making", we have highlighted the work of Pearson et al. and Devapriya et al. with StratBAM, as examples of decision-making within the scope of our model. These studies utilized LOS models to estimate the need for resources, such as Intensive Care Beds, across various DRGs. That being said, we have also emphasized that statistical models for LOS, such as the one we propose, represent just one component of hospital capacity planning software. Such planning also demands the consideration of other critical factors. Thus, we additionally define the scope of our work with less ambiguity with "It is this specific aspect - the decision of the statistical model to represent LOS - that our present paper primarily addresses."

Further, the various graphical tools we have proposed could be employed to dynamically determine thresholds and use them for long-stay patient reviews.

I consider further the managerial implications. The statements lines 708-721 about LOS seem fully accurate. However, I do not appreciate that the authors have shown that their new mathematics was necessary for lines 708-721. In other words, the facts seem accurate, and they are managerial implications of LOS being skewed. However, please add PRECISE information about how SPECIFICALLY these implications are from the authors' new results, as compared with knowledge about LOS beforehand.

We emphasize that study does not only propose a model for decision-making - the beta-geometric distribution discussed in Section "4.5. Modeling Length of Stay," but also offers a detailed analysis of the inherent properties of empirical Length of Stay (LOS) using Extreme Value Theory, a well-established area of statistics. Our study is, for instance, in line with Baek,

H., Cho, M., Kim, s., Hwang, H., Song, M., Yoo, S. Analysis of length of hospital stay using electronic health records: A statistical and data mining approach. PLoS One, 13(4), 2018 in studying the fundamental statistical properties of LOS, on a different scale, and Bonetti, M., Cirillo, P., Musile Tanzi, P., Trinchero, E. An Analysis of the Number of Medical Malpractice Claims and Their Amounts. PLoS ONE 11(4): e0153362, 2016. doi:10.1371/journal.pone.0153362, utilizing Extreme Value Theory.

We have abridged the “Managerial Implications” section and revised it to underscore, not only the obvious skewness of LOS, but the heavy-tailedness of its distribution. In practical terms, this implies that outlier observations, often viewed as surprising or exceptional in the clinical context, are in fact relatively common and constitute a significant portion of the overall distribution. This is a crucial finding that addresses observations made by clinicians and in studies such as Hughes et al., 2021, where prolonged stays are perceived as exceptional.

In essence, our contribution seeks to recalibrate these intuitive expectations, which are often based on the assumptions of a 'Normal' distribution, to reflect the actual statistical properties of LOS.

Regarding pricing, the authors refer to revenue management. Please provide DETAILS of PRECISELY what decisions the authors are suggesting. The authors know LOS and charges in retrospect, so how is this revenue management? For revenue management, that’s prospective, before the product is purchased. The “revenue” in the authors’ context is to be obtained from whom and when? Furthermore, at least for surgery as I know, the models regarding hospital financial decision-making long-term incorporate other variables than LOS and “charges,” specifically the indirect/intangible value (“utility”) associated with individual types of patients. For example, suppose that there were low or negative contribution margin for vascular surgery because of long LOS outliers. That does not mean that one can remove vascular surgery, because without vascular surgeons, there can be no obstetrics, general surgery, urology, trauma, etc., at the hospital. Hospitals generally need all specialties because the patients and the complications involve multiple specialties. This is why without the authors referring to SPECIFIC decisions with DETAILS from other studies, I recommend that the authors markedly reduce the background sections, implications sections, etc., and simply present their (very) reasonable methods and results.

We appreciate your constructive feedback and agree with your points regarding the complexity of hospital revenue management. Given this complexity, we've revised our manuscript to focus solely on our main contributions, namely, identifying properties of length of stay (LOS) using Extreme Value Theory, and proposing the beta-geometric distribution for modeling LOS.

In response to your comments, we've withdrawn certain conjectures related to revenue management that weren't directly supported by our research. We have kept our discussion

focused on our key findings based on empirical data from Siriraj Hospital (i.e. the observation that long stays are currently underpriced and their opportunity cost compounds).

Thank you for your valuable insights that have helped us refine our study and improve the precision of our presentation.

Reviewer #2: Dear authors,
In my opinion the paper is much more a statistical paper than a health sciences/ public health paper; the structure is still not adequate; please dont include all the mathematical equations (they are very usefull for statistitians, but not for the most of the public health lectors)
The authors must include less statistics methods and more public health discussion and more relevance on their approach to the public health field.
We appreciate your feedback and understand your perspective. However, our paper does not claim to have relevance for public health. It is fundamentally rooted in Operations Management and focuses on the mathematical properties of Length of Stay (LOS) as a random variable. As such, it is primarily intended for operations researchers and data science within healthcare.

For these reasons, the keywords associated with our manuscript are “Length of Stay, Statistical Modeling, Extreme Value Theory, Beta-Geometric Distribution.”

Recognizing your concerns, we have revised our manuscript to clearly state this scope in the Decision-making section, helping to clarify the audience and context for our work. Furthermore, we've made an effort to limit conjectural statements about broader health implications and to focus more on our core results, in order to avoid any confusion regarding the scope and intent of our paper.

We trust that these changes align with your insightful comments and hope that they help clarify the nature and intent of our research.
