# Supplementary material for: Hospital length of stay: A cross-specialty analysis and Beta-geometric model
Source: PLoS One. 2023 Jul 13;18(7):e0288239. doi: 10.1371/journal.pone.0288239 (PMC10343164; doi:10.1371/journal.pone.0288239)
Supplement: Attachment — Submitted filename: Response to Reviewers.docx [file pone.0288239.s013.docx]

Original Manuscript ID: PONE-D-21-29331

Original Article Title: “Hospital Length of Stay: A cross-Specialty Analysis and Beta-Geometric Model”

To: PLOS One Editor

Re: **Response to reviewers**

Dear Editor,

Thank you for allowing a resubmission of our manuscript, with an opportunity to address reviewers comments.

We are uploading (a) our point-by-point response to the review comments (below) (response to reviewers), (b) an updated manuscript with track changes, and (c) a clean updated manuscript without track changes.

1. Please ensure that your manuscript meets PLOS ONE's style requirements, including those for file naming. The PLOS ONE style templates can be found at

<https://journals.plos.org/plosone/s/file?id=wjVg/PLOSOne_formatting_sample_main_body.pdf> and

<https://journals.plos.org/plosone/s/file?id=ba62/PLOSOne_formatting_sample_title_authors_affiliations.pdf>

Thank you, we have updated the manuscript and file names accordingly.

2. In ethics statement in the manuscript and in the online submission form, please provide additional information about the patient records used in your retrospective study. Specifically, please ensure that you have discussed whether all data were fully anonymized before you accessed them and/or whether the IRB or ethics committee waived the requirement for informed consent. If patients provided informed written consent to have data from their medical records used in research, please include this information.

The ethics statement has been updated and a statement about IRB approval and the anonymization of patient data has also been made in Section 5. Methods.

3. In your Data Availability statement, you have not specified where the minimal data set underlying the results described in your manuscript can be found. PLOS defines a study's minimal data set as the underlying data used to reach the conclusions drawn in the manuscript and any additional data required to replicate the reported study findings in their entirety. All PLOS journals require that the minimal data set be made fully available. For more information about our data policy, please see <http://journals.plos.org/plosone/s/data-availability>.

A dataset recording lengths of stay in the four medical specialty departments considered will be uploaded as supplemental data with this submission.

4. Please include your full ethics statement in the ‘Methods’ section of your manuscript file. In your statement, please include the full name of the IRB or ethics committee who approved or waived your study, as well as whether or not you obtained informed written or verbal consent. If consent was waived for your study, please include this information in your statement as well.

The ethics statement has been updated and a statement about IRB approval and the anonymization of patient data has also been made in Section 5. Methods.

5. Please ensure that you refer to Figures 13, 19, and 20 in your text as, if accepted, production will need this reference to link the reader to the figure.

Thank you. All figures in the Appendix have been referred to in the body of the revised manuscript.

6. Please include captions for your Supporting Information files at the end of your manuscript, and update any in-text citations to match accordingly. Please see our Supporting Information guidelines for more information: <http://journals.plos.org/plosone/s/supporting-information>.

Captions have been added to supporting information in the revised manuscript.

#1) The authors appropriately refer to individual DRG (line 134) and to decision-making (line 116). I think that the authors need to list several specific decisions that the authors are considering, not analyses per se but literally decisions. Consider which ones do not involve analyses by DRG and therefore are addressed by the current paper, because most of the decisions that I know about would be by DRG (e.g., guiding individual patients) or by knapsacks/ collections of DRGs (e.g., comparing hospitals conditional on the relative distributions of different DRGs). This matters because the authors currently are not performing analyses by DRG. If most decisions would be by DRG, then the authors’ analyses should be by DRG.

Thank you very much for raising this important point, allowing us to more precisely delimit the decision-making scope of the proposed statistical model. The brief answer to the above question is Capacity Planning; that is operational decisions about resources that are shared across multiple DRGs (hospital beds, vehicles, equipment, non-specialist workforce).

A recent (April 2022) literature review on Capacity Planning [1] lists the main ones of these decisions, as follows:

- How many ward beds should be allocated for different specialties?
- How to align the hospital configuration to forecasted patients volumes?
- What proportion of time should be allocated to different specialties in operating theaters?

We agree that general mentions of “decision-making” in the initial manuscript were too broad and may be interpreted as including clinical decision-making.

Following this comment, Section 2. “Decision-Making Scope” has been added to the revised manuscript, and several additional references pertaining to capacity planning and the need for more refined models of LOS for operational decision-making have been compiled and referenced in this section. We have also revised the “Introduction” and “Managerial Implications” sections, and avoided the use of “decisions” and “decision-making” with more precise mentions of “capacity planning”.

Indeed, models of length of stay (LOS) by DRG are useful for clinical decision-making or quality/performance evaluation, as kindly raised by Reviewer #1 (guiding patients, quality/performance comparison), and mentioned in lines 58-64 of the initial manuscript. However, as discussed in the newly created “Decision-Making Scope” Section, making capacity decisions for shared resources requires simulating LOS volumes for all DRGs within a healthcare unit by fitting a theoretical distribution on empirical data (as is performed in the “Modeling Length of Stay” section), which also requires estimating the empirical moments of the distribution (mean, variance, skewness, kurtosis, etc.), among other input parameters.

This distinction between clinical decisions and capacity planning was in fact at the inception of our multidisciplinary team (covering clinical, surgery, operations, and finance specialties) and this paper.

This work originated from our observation that statistical models of LOS by DRG, which are useful for guiding patients and making clinical decisions did not “scale up” to good models for capacity planning at Siriraj Hospital. As discussed in the paper, the main limitation in aggregating several (thin-tailed) statistical models by DRG is that outliers (very long stays) appear less likely and less significant. These outliers (which are only considered as such because of the thin-tailed nature of the models) have in fact a disproportionate impact on resource consumption (as the Section “On the concentration of LOS” shows).

Our observations have since been echoed by a recent publication that appeared since the submission of this manuscript. Indeed, [2] observes that “Outlier cases utilize a disproportionate and increasing share of hospital resources and available beds. The current tendency to exclude such outlier stays in data reporting due to assumed rare occurrence may need to be revisited”, and [3] note that “Neither are diagnosis-related groups an appropriate methodology for capacity planning …This means that they can be used for calculating prices but they say little about the mix of resources that is needed.”

The authors write at line 704 “it offers a consistent model for decision-support”. Please provide those decisions.

At line 706 “can also be scaled down to individual DRGs.” Without being scaled down, for what decisions, and if needs to be scaled down, I would think that confirm results and usefulness when scaled down.

Section 2. “Decision-Making Scope” of the revised manuscript hopefully clarifies these decisions. Moreover, we have replaced the general use of the term “decision-support” (which can indeed include clinical decision-making), by the more precise term “capacity planning”.

1a. For example, from line 119 “if the goal of the analysis … is hospital comparison, uniform trimming of LOS across all hospitals might be inappropriate.” My concern is that “might” is vague and the authors are not providing specific analyses of specific decisions. Under many conditions trimming works. See, for example, the following paper and its references: “Proportions of Surgical Patients Discharged Home the Same or the Next Day Are Sufficient Data to Assess Cases' Contributions to Hospital Occupancy,” Cureus 2021 doi: 10.7759/cureus.13826. Surely the authors’ modeling focused on long LOS is different than that paper. However, without the authors considering several specific decisions, the reliability of the authors’ claims seem limited.

Thank you, the reference you kindly provided offers an excellent illustration for the distinction between clinical decisions for individual cases and capacity planning at the level of a healthcare unit made in the revised manuscript. We have added this reference and contrasted it with the capacity planning models discussed in Section 2. “Decision-Making Scope”.

Please note that the decision in the reference kindly provided by reviewer #1 is clinical and concerns individual patients (“which cases may need to be postponed”). At an operational, larger scale, however, [4] observes that “outlier cases utilize a disproportionate and increasing share of hospital resources and available beds”. In our model, these are not outliers, but an essential component of the distribution (the tail) that determines many of its statistical properties.

1b. For example, the authors’ references to charges seem very likely to be dependent on individual payment systems (e.g., Line 507 “up to a certain threshold … patients are approximately charged the same amount per day”). Normally I would think that a suitable approach would be to have analyses of charges in supplemental content or a separate section specific to the authors’ country. However, with the authors not referring to specific decisions, I am unable to make a specific recommendation. The authors need please to be modeling a few (e.g., 2 or 3) specific decisions that are common and necessary.

Thank you for this comment. We have added details about the payment system in Thailand explaining the opportunity cost of long stays (payments are largely determined by DRGs, co-morbidities, and procedures performed, independently of LOS). We have also found these observations to be aligned with those made in the payment system of the USA (Medicare) by [4], and added a comparison in the same section. Please note that the purpose of the Section “On revenue” is to highlight the opportunity cost of long stays comparatively to shorter ones, which can be observed independently of the payment (although it is explained by it).

1c. For example, “second moments (variance) … show a slow convergence” (line 569). Provide please specific common, important, decision pooling among DRGs for which one would want to estimate the variance. Referring to my first point, hospitals differ in their relative distributions of DRGs, and yet the authors have not modeled by DRG. Therefore, what is the decision? From line 574 “this problem is particularly acute for LOS data per patient in surgery.” Why would one be doing this, to decide or judge what?

A simple usage of the variance (square of the standard deviation) is to measure the standard error for the mean LOS (given by).

A slow converging variance/standard deviation means that it requires very large samples to be accurately measured.

Second moments (variance) is also the basis of least squares regression. As discussed in the “Managerial Implications”section” This catastrophe principle, inherent to subexponential random variables, makes models for forecasting precise values of LOS (e.g. least square

regressions based on second moments) inapplicable.”

Besides these, fitting distributions to empirical data for the purpose of capacity planning requires estimating the empirical moments of the variable at hand (mean, variance, skewness, kurtosis). The max to sum plots presented in the paper can be used to determine the appropriate sample sizes for the empirical moments to converge to their “true” theoretical value.

#2)

Table 3 Gini index please add some standard errors, confidence intervals, equivalent to show that the indices are estimated sufficiently precisely given the sample sizes such that comparisons among them are appropriate. The same applies to the correlations in Table 4. Assure that discussion refers only to differences that are reliably (significantly) different correlations.

Thank you. Standard errors and confidence intervals for both statistics have been computed and included in the revised manuscript, with a reference to the method used for the Gini coefficient (bootstrap re-sampling in R).

#3)

MINOR issues of writing

P1 abstract “would, counter-intuitively”. I do not follow why this is counter-intuitive. Consider deleting the phrase and just stating the behavior. Same line 599.

Thank you. We agree that the term subjectively assumes prior knowledge of the reader, and it has been removed in the revised manuscript.

The intended meaning was that the observed behavior (“the longer a patient has been admitted, the higher their expectation of *remaining* admitted”, corresponding to the Lindy Effect <https://en.wikipedia.org/wiki/Lindy_effect>) goes against a layman’s expectation for the completion of a task (we expect something to complete sooner, the longer it has been going on), and what a thin-tailed model (Gaussian, Poisson, etc.) would produce.

Line 383 “notoriously”, I do not follow what is the matter with the behavior. Why is this word needed? Consider deleting.

Thank you. This term has been replaced with “is known to”, which was its intended meaning.
